# Effects of spironolactone on extrasystoles and heart rate variability in haemodialysis patients: a randomised crossover trial

**DOI:** 10.48101/ujms.v126.5660

**Published:** 2021-01-25

**Authors:** Michael Eklund, Olof Hellberg, Hans Furuland, Yang Cao, Erik Nilsson

**Affiliations:** a Department of Internal Medicine, School of Medical Sciences, Örebro University, Örebro, Sweden; b Department of Medical Sciences, Uppsala University Hospital, Uppsala, Sweden; c Clinical Epidemiology and Biostatistics, School of Medical Sciences, Örebro University, Örebro, Sweden; d Unit of Integrative Epidemiology, Institute of Environmental Medicine, Karolinska Institutet, Stockholm, Sweden; e Department of Medical Epidemiology and Biostatistics, Karolinska Institutet, Stockholm, Sweden; f School of Medical Sciences, Örebro University, Örebro, Sweden

**Keywords:** Haemodialysis spironolactone, clinical trial, long-term ECG, arrhythmias, heart rate variability

## Abstract

**Background:**

Spironolactone treatment reduces mortality in haemodialysis (HD) patients. The objective of this study was to evaluate if spironolactone affects cardiac electric activity in this population.

**Methods:**

Participants were randomised to start with spironolactone 50 mg daily or observation (12 weeks) with subsequent washout (6 weeks) and crossover to the other intervention (12 weeks). Long-term electrocardiograms were recorded and assessed with blinding to treatment. The primary outcome was premature ventricular complexes (PVC), and secondary outcomes were atrial premature contractions (APC) and heart rate variability (HRV).

**Results:**

Thirty participants were recruited, and data for 16 participants were included in the analysis. Treatment was associated with an increase in PVCs by 9.7 [95% confidence interval (CI): 1.5 to 18] h^−1^. HRV time-domain variables increased during treatment, the standard deviation of all beat-to-beat intervals by 18 (95% CI: 3.3 to 32) milliseconds (ms) and the standard deviation of the averages of beat-to-beat intervals in all 5-min segments of the entire recording by 16 (95% CI: 1.5 to 30) ms. There were no significant differences in other variables.

**Conclusion:**

Spironolactone treatment increases PVCs in HD, indicating a possible proarrhythmic effect. However, improved cardiac autonomic function, as indicated by an increased HRV, may contribute to the survival benefit from spironolactone treatment in HD patients.

## Introduction

Cardiovascular disease is a leading cause of morbidity and mortality in end-stage renal disease (ESRD) (1). In two recent trials, treatment with the mineralocorticoid receptor antagonist (MRA) spironolactone improved survival and reduced the risk of cardiovascular events in haemodialysis (HD) patients (2,3). However, the mechanisms by which MRAs may improve survival in HD patients are not clear.

Multiple studies suggest a link between the mineralocorticoid aldosterone and cardiac electric activity (4–6). Animal studies show that overexpression of the mineralocorticoid receptor in cardiac myocytes increases the occurrence of ventricular arrhythmias (6). It also reduces heart rate variability (HRV) (6), which is a marker for autonomic function (7). In line with this, MRAs decrease atrial arrhythmias in rodents (5). A reduction in HRV, as well as the presence of premature ventricular complexes (PVCs) and atrial premature contractions (APCs), has been associated with an increased risk of death due to cardiovascular events (8–11). These electrocardiogram (ECG) markers are also associated with the risk of arrhythmias (10,12–14).

This study aimed to investigate the effects of MRA on cardiac electrical activity in HD patients. We hypothesised that spironolactone treatment would be associated with a change in the number of PVCs on long-term ECG (LTECG).

## Materials and methods

This was an open-label, randomised crossover trial controlled using untreated observation with blinded assessment of outcomes. The primary outcome was the frequency of PVCs, and secondary outcomes were the frequency of APCs and change in HRV indices.

Patients were recruited in Sweden, from the dialysis units at Örebro University Hospital and Uppsala University Hospital, from February 2013 until December 2014. Inclusion criteria were age 18 years or older, three or more weekly HD-sessions, compliance during the previous three months and adequate cognitive function. Exclusion criteria were cardiac pacemaker, persistent or permanent atrial fibrillation, plasma potassium > 6.5 mmol/L at any time during the two months preceding inclusion, life-expectancy of less than 12 months, and pregnancy or breastfeeding.

Power calculation was based on PVCs and yielded a minimum of 14 subjects completing the crossover study. The analysis was based on results from trials in congestive heart failure (15–17) and used a two-sided significance level of 5% and 80% power. The sample size was set to 30 as a dropout rate of 50% was expected, mainly due to renal transplantation, mortality and side effects of spironolactone.

Randomisation and allocation were conducted by a person not otherwise involved in the study. Sequence generation in blocks of six with a ratio of 1:1 was performed using Clinstat (Martin Bland, United Kingdom). Sequentially numbered, sealed and opaque envelopes were used for allocation.

Subjects were assigned to a sequence of 12 weeks 50 mg oral spironolactone daily, followed by 6 weeks washout phase and finishing with 12 weeks of no treatment, or the reverse order. Spironolactone and its active metabolites have half-lives of 1.4–16.5 h in healthy individuals, but the effects of chronic kidney disease and HD on the elimination of spironolactone are unknown (18). The lack of knowledge on the elimination of spironolactone in the study population motivated the use of a long washout phase.

The four study visits took place on midweek dialysis sessions and were conducted before and after both of the 12-week intervention periods. Blood draw and measurements were performed before dialysis treatment. Blood pressure was measured using an automated oscillometric blood pressure monitor (Omron M6 AC, Omron Healthcare, Japan). The accredited local hospital laboratories analysed routine biochemical parameters. LTECG recordings were started at the onset of the dialysis session and continued for a minimum of 24 h, using a Medilog AR12 Plus ECG recorder (Schiller AG, Switzerland). The Swedish Renal Registry was accessed to obtain information on the primary renal disease of the participants.

A clinical laboratory scientist and a physician, blinded to treatment allocation, curated the LTECG recordings, using Medilog Darwin 2 Enterprise v 2.1.0 software (Schiller AG, Switzerland). HRV analysis was conducted using R-statistics v 3.4.1 (R Development Core Team, Austria) and the package RHRV v 3.0.7 (19). HRV variables were defined in accordance with Malik et al. (7) as follows: Time-domain variables included the standard deviation of all beat-to-beat intervals (SDNN), the standard deviation of the averages of beat-to-beat intervals in all 5-min segments of the entire recording (SDANN) and the square root of the mean of the sum of the squares of differences between adjacent beat-to-beat intervals (RMSSD). Frequency-domain variables were based on power in different frequency ranges, 0.15–0.4 Hz – high frequency (HF), 0.04–0.15 Hz – low frequency (LF), 0.003–0.04 – very low frequency (VLF) and ≤ 0.003 Hz – ultra-low-frequency (ULF).

After treatment, remaining spironolactone tablets were collected. Compliance was calculated as the ratio of tablets consumed, based on the amount returned, to expected consumption.

Baseline data were expressed as median and interquartile range, or as percentage. A generalised linear mixed model with random patient effects was used to assess the treatment effect in all statistical analyses, as described by Senn (20). Period effects and baseline values were considered fixed effects. Log transformation to normality was used when mandated by the assessment of model residuals. All analyses were conducted using R-statistics v 3.4.1 (R Development Core Team, Austria). Per-protocol analysis with listwise deletion of incomplete cases was pre-specified in the study analysis plan, motivated by the study aim of elucidating MRAs mechanisms of action rather than to assess clinical efficacy.

The study received ethical approval by *The Regional Ethical Review Board in Uppsala*, Sweden (reference number 2011/316). The board decision covers both study centres of this trial. All participants gave written informed consent before enrolment. The Declarations of Helsinki was adhered to, and Good Clinical Practice was applied. The trial was approved by the Swedish Medical Products Agency (EudraCTnr 2011-002773-39) and was registered on 28 September 2011 at http://www.clinicaltrialsregister.eu/ (EudraCTnr 2011-002773-39). Monitoring was performed to ensure adherence to the study protocol, complete documentation and complete registration of adverse events.

## Results

### Baseline characteristics

**Figure 1:**
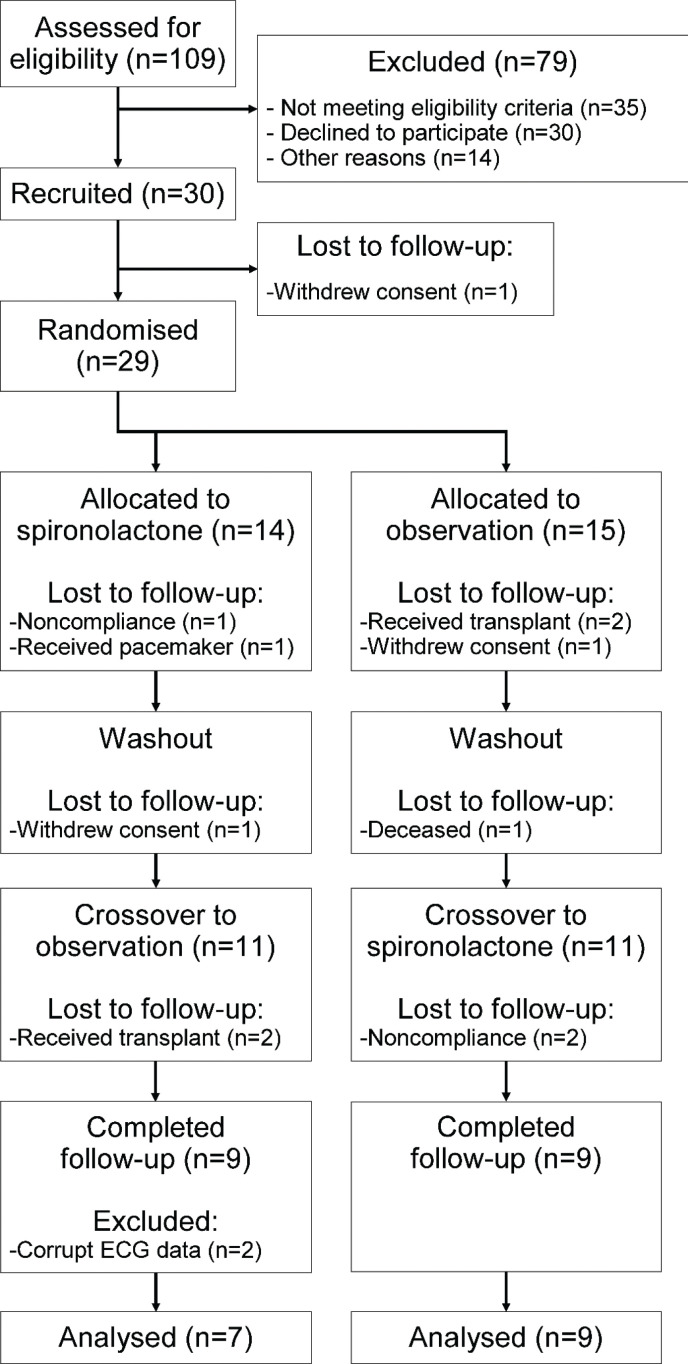
Flow chart of the study procedure. Study design, recruitment of participants and follow-up for long-term electrocardiogram data.

A total of 30 participants were recruited, of which 18 (60%) were enrolled in Örebro and 12 (40%) in Uppsala. Of these, 18 persons (60%) completed follow-up (Figure 1). There was a larger proportion of participants completing follow-up in Örebro [13, (72%)] than in Uppsala [5, (42%)]. Two participants had corrupt LTECG data files from single recordings and were excluded, resulting in a total of 16 participants (53%) included in analysis. Of the analysed population, the median age was 66 (61.5–67) years, and 12 (75%) were male. Only two (13%) of the included participants were current tobacco users, and three (19%) participants were previous tobacco users. Eight participants (50%) were treated with angiotensin-converting enzyme inhibitors or angiotensin receptor blockers (Table 1). Further, eight persons (50%) were treated with a beta-adrenoceptor antagonist. No other class of anti-arrhythmic medication was used. The potassium binder polystyrene sulphonate was used at inclusion in two cases (12%), and one participant (6.2%) was treated with potassium chloride supplementation. Concomitant medications have been listed in Supplementary Table 1.

**Table 1:** Baseline characteristics.

Variable					
**Comorbidities**	**Number**	**%**	**Dialysis data**	**Median**	**Interquartile range (IQR)**
*Cardiovascular*	*8*	*50*	Time in dialysis, days	480	1,000
Myocardial infarction	4	25			
Angina pectoris	6	38	**Physiological data**	**Median**	**IQR**
Congestive heart failure	4	25	Heart rate, min^−1^	76	11
Arrhythmia	2	12	Systolic blood pressure, mmHg	140	30
Peripheral vascular disease	3	19	Diastolic blood pressure, mmHg	72	18
Stroke	3	19	*Extra systoles*		
*Diabetes mellitus*	*7*	*44*	Premature ventricular complex, h^−1^	2.7	7.2
Diabetes mellitus, type 1	1	6.2	Atrial premature contraction, h^−1^	4	21
Diabetes mellitus, type 2	6	38	*Heart rate variability (HRV) Time-domain*		
*Other comorbidities*			Standard deviation of all beat-to-beat intervals (SDNN), milliseconds (ms)	110	43
Dyslipidaemia	11	69	Standard deviation of the averages of beat-to-beat intervals in all 5 min segments of the entire recording (SDANN), ms	92	42
Obstructive sleep apnoea syndrome	5	31	Square root of the mean of the sum of the squares of differences between adjacent beat-to-beat intervals (RMSSD), ms	22	33
			HRV Frequency-domain*		
**Biochemical data**	**Median**	**IQR**	High frequency (HF), ms^2^	28	100
Potassium, mmol/L	4.8	0.76	Low frequency (LF), ms^2^	100	120
Sodium, mmol/L	140	2.2	Very low frequency (VLF), ms^2^	220	250
Magnesium, mmol/L	0.9	0.13	Ultra-low-frequency (ULF), ms^2^	50	26
Calcium, mmol/L	2.3	0.18			
Bicarbonate, mmol/L	23	2.8			
Phosphate, mmol/L	1.6	0.63			
Albumin, g/L	38	3			
Haemoglobin, g/L	120	18			
C-reactive protein, mg/L	3.5	12			
Parathyroid hormone, pmol/L	140	210			

Baseline characteristics of the 16 persons included in long term electrocardiogram analysis, reported as number and percentage or as median and interquartile range.

* Power in different ranges, HF 0.15–0.4 Hz, LF 0.04–0.15 Hz, VLF 0.003–0.04 Hz, ULF ≤0.003 Hz.

### Outcomes

**Figure 2:**
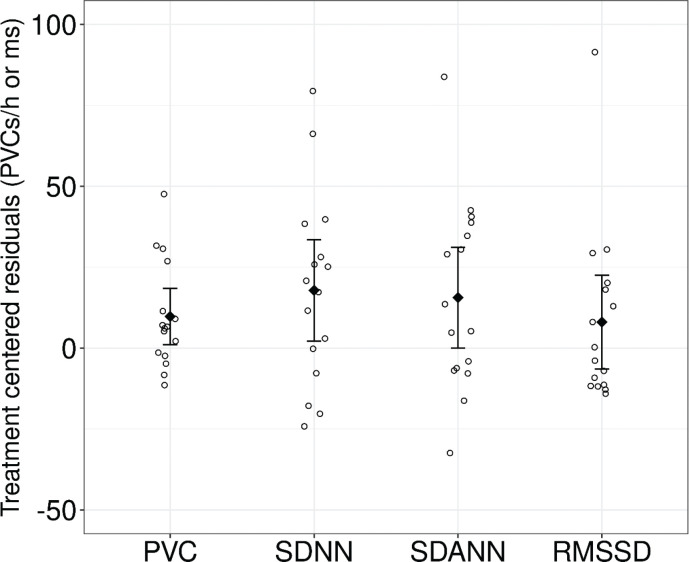
Effect of spironolactone on premature ventricular complexes and heart rate variability time-domain. Treatment centred residuals were calculated from the generalised linear mixed model with random effects residuals, which was added to treatment estimates, representing both treatment effect and random influences not eliminated by study design or adjustment. Diamonds signify means and horizontal bars 95% confidence intervals. Individual values are plotted as circles. Abbreviations: ms, milliseconds; PVC, premature ventricular complexes; RMSSD, the square root of the mean of the sum of the squares of differences between adjacent beat-to-beat intervals; SDANN, the standard deviation of the averages of beat-to-beat intervals in all 5-min segments of the entire recording; SDNN, the standard deviation of all beat-to-beat intervals.

The 16 participants included in the analysis had a mean compliance of 97%. Spironolactone treatment was associated with an increase in PVCs compared to observation [mean treatment effect 9.7, 95% confidence interval (CI): 1.5 to 18 h^-1^]. There was no significant difference in APCs during treatment compared to observation (ratio 2.5, 95% CI: 0.58 to 11). All HRV time-domain variables (SDNN, SDANN, RMSSD) increased during treatment, although statistical significance was reached only for SDNN, which increased by 18 (95% CI: 3.3 to 32) milliseconds (ms), and SDANN, which increased by 16 (95% CI: 1.5 to 30) ms (Table 2). The effects of spironolactone on these variables on the individual level are presented in Figure 2. HRV frequency-domain variables (HF, LF, VLF, ULF) did not change significantly during treatment compared to observation (Table 3). Note that in Tables 2 and 3, the LTECG variables measured at the end of the spironolactone treatment period were compared to the measurement after the observation period, and since the estimate was adjusted for both baselines, it corresponds to the change in each variable that is due to spironolactone treatment.

**Table 2:** Effect of spironolactone on premature ventricular complexes and heart rate variability time-domain variables.

Variable	Estimate	95% confidence interval	*p*
Premature ventricular complex, h^-1^	9.7	1.5 to 18	0.024
Standard deviation of all beat-to-beat intervals (SDNN), milliseconds (ms)	18	3.3 to 32	0.02
Standard deviation of the averages of beat-to-beat intervals in all 5-min segments of the entire recording (SDANN), ms	16	1.5 to 30	0.033
Square root of the mean of the sum of the squares of differences between adjacent beat-to-beat intervals (RMSSD), ms	8	−5.7 to 22	0.23

Estimates of the treatment effect of spironolactone, corresponding to absolute change in the respective variable. Estimates, confidence intervals and *p*-values were calculated from a generalised linear mixed model with random effects, using fixed effects for treatment, intervention order and baseline values as well as random effects for patients.

**Table 3:** Effect of spironolactone on heart rate variability frequency-domain variables.

Variable*	Estimate	95% confidence interval (CI)	*p*
High frequency (HF)	1.8	0.81 to 4.1	0.13
Low frequency (LF)	1.3	0.77 to 2.3	0.29
Very low frequency (VLF)	1.3	0.94 to 1.7	0.11
Ultra-low frequency (ULF)	1.2	0.83 to 1.6	0.35

Estimates of the treatment effect of spironolactone, corresponding to the ratio between treatment and observation. Values were log-transformed, and estimates, confidence intervals and *p*-values were calculated with a generalised linear mixed model with random effects. Treatment, intervention order and baseline values were used as covariates, and patient effects were treated as random. Estimates and confidence intervals were antilogged to get ratios.

* Power in different ranges, HF 0.15–0.4 Hz, LF 0.04–0.15 Hz, VLF 0.003–0.04 Hz, ULF ≤0.003 Hz.

During the study period, the following changes in anti-arrhythmic treatment were made: Three participants initiated beta-blocker treatment (bisoprolol in all cases), three discontinued treatment (two cases of atenolol, and one case of metoprolol) and three had a reduction in dose (bisoprolol in one case, and metoprolol in two cases). All changes, except a dose reduction of metoprolol for one participant, occurred during the non-intervention period of follow-up.

There was no apparent effect of spironolactone on plasma potassium concentrations, with an estimated mean difference in plasma potassium between the end of treatment and the end of observation of −0.046 mmol/L (95% CI: −0.57 to 0.47). Potassium binder polystyrene sulphonate was initiated for one participant during spironolactone treatment, and no other changes in potassium binder treatment occurred during follow-up. Serum bicarbonate concentration decreased by −2.0 (95% CI: −3.5 to −0.52) mmol/L during treatment. Effects on electrolytes have been presented in Supplementary Table 2.

There were no changes in systolic blood pressure (13 mmHg, 95% CI: −1.8 to 28) or diastolic blood pressure (5.6 mmHg, 95% CI: −1.1 to 12) during treatment compared to observation (Supplementary Table 2).

During the study, 62 adverse events were reported for the study population that underwent at least one study visit (*n* = 29). One serious adverse event was reported, with a participant passing away during the wash-out period of follow-up. The case review did not indicate any association between death and exposure to the study drug. One case of muscular cramps was considered associated with spironolactone treatment. All other events were judged as not being associated with the intervention. The most common adverse events were infections (12 occurrences), chronic obstructive pulmonary disease exacerbations (10 occurrences) and hypotension (four occurrences, of which two were during spironolactone treatment).

## Discussion

The major novel findings of this study were that spironolactone treatment is associated with an increased occurrence of PVCs and that it increases HRV time-domain indices in persons undergoing HD. Although an increase in PVCs during treatment was unexpected, effects on ECG variables are in line with mineralocorticoid receptor action on cardiac electrical conductivity (4–6). Spironolactone has been shown to act as a partial mineralocorticoid receptor agonist during certain intracellular conditions in vitro (21, 22), and we speculate that the relatively high dose of spironolactone used in this study may have contributed to an increased occurrence of PVCs, thus having a proarrhythmic effect. HRV reflects autonomic function (7), and the observed increase in HRV may be due to inhibition of aldosterone action in the central nervous system, leading to a reduction in cardiac sympathetic stimulation, as has been shown in animal studies (23). In individuals with congestive heart failure, sympathoexcitation has been associated with a reduction in HRV (24) and increased risk of death (25). Increased HRV, as demonstrated here, could, therefore, be a mechanism underlying the beneficial effect of MRA in HD patients (2, 3).

We also found an association between spironolactone treatment and a decrease in plasma bicarbonate concentration, which may be due to aldosterone action on Na^+^/H^+^ exchange in the intestine (26) or remaining nephrons, with secondary effects on bicarbonate reabsorption.

Anti-arrhythmic effects of spironolactone have been suggested as an important mechanism of action leading to improved survival in congestive heart failure and ischemic heart disease, reducing the occurrence of PVCs (16, 27, 28) and APCs (16). To our knowledge, no prior studies have investigated the effect of spironolactone on these markers in ESRD. In contrast to studies on heart disease patients (16, 27, 28), we found that MRA treatment increased the frequency of PVCs and did not demonstrate an effect on APCs. Differences in serum potassium concentrations (29), cardiovascular pathology and concomitant medications between these populations are possible explanations for these discrepancies. For example, in this study, only 25% of the participants had congestive heart failure, and 25% had a history of myocardial infarction, compared to 50–52% with previous myocardial ischemia in the congestive heart failure population (16). Furthermore, previous studies in chronic heart disease patients reported the use of angiotensin-converting enzyme inhibitors in more than 90% of cases (16, 27), compared to 50% using angiotensin-converting enzyme inhibitors or angiotensin receptor blockers in our study. These differences could indicate that a beneficial effect of spironolactone on APCs and PVCs is dependent on pathological processes that are less prevalent in HD patients than in congestive heart failure and ischemic heart disease, or that the effect requires a more extensive renin–angiotensin–aldosterone system inhibition, achieved through concomitant angiotensin-converting enzyme inhibitors treatment.

A few trials have investigated the effect of spironolactone on HRV in ESRD. In a study by Flevari et al. (30), 14 HD patients without congestive heart failure were treated with spironolactone 25 mg three times weekly for 4 months. In line with our results, the authors found that HRV increased in the time-domain variable RMSSD during treatment but also that HRV frequency-domain indices (ULF and VLF) decreased. Effects on SDNN, the most commonly used marker for cardiac autonomic function, were not reported. Lin et al. (2) conducted a trial with 253 ESRD patients, who received spironolactone 25 mg daily, or placebo, for two years. Contrary to our results, spironolactone treatment did not affect HRV variables, and although Lin et al. had a larger sample size than our study, differences in dosing and treatment duration should be noted. In congestive heart failure, and consistent with our results, spironolactone 50–100 mg daily increased SDNN, SDANN (31) as well as HF and LF/HF (32) after 4–8 weeks of treatment. Although results from ESRD patients are not consistent, an increase in HRV has been found in multiple studies in different populations, indicating a beneficial effect of spironolactone on cardiac autonomic function as measured by HRV.

Contrary to our expectations and previous findings by Flevari et al. (30), we did not observe blood pressure reduction with spironolactone, but rather a slight but not statistically significant increase. It should be noted that the participants in the study by Flevari et al. had more extensive use of antihypertensive agents at baseline, and a slightly longer intervention period (4 months), which may explain the differences.

The use of MRAs in individuals with reduced renal function is limited by the risk of hyperkalemia (33). In this study, we observed no cases of severe hyperkalemia, and the treatment did not affect mean plasma potassium concentration. Multiple studies of spironolactone in ESRD have been conducted, each one individually not showing an increased risk of severe hyperkalemia in spironolactone treatment, although a recent meta-analysis weighted estimate indicates that the risk is increased (34). In consequence, and despite the lack of effect on potassium in this study, the risk of hyperkalemia during spironolactone treatment in ESRD should be recognised and appropriate monitoring conducted.

Some limitations apply to this study. Although the number of patients analysed was sufficient based on the power analysis, our sample size was limited. However, the crossover design increases statistical power, and we emphasise that according to the power calculation performed before study start, the analysis has sufficient power for detecting relevant changes in the primary outcome of PVCs. For secondary outcomes, the sample size may have been too small to show relevant effects, although it should be noted that all HRV variables measured showed a trend towards increasing values after treatment, which is concordant with the finding of statistically significant increases in SDNN and SDANN. Further, we did not use placebo during the control period, which allows for the theoretical possibility of an anticipation effect among participants. However, it is unlikely that such an effect would affect ECG variables and we employed blinded assessment of ECG recordings to avoid assessment bias. An additional limitation is that only four participants were female, and results could therefore not be analysed separately by sex. We also acknowledge the multiplicity of analyses and emphasise that change in PVCs was postulated as the primary endpoint in this study and that all analyses were specified *a priori*. Strengths of the study include treatment randomisation, blinded assessment of outcomes and the crossover design, which limits confounding and increases statistical power. Also, this study included HD patients with and without congestive heart failure and may, therefore, be valid for a relatively unselected HD population.

We conclude that improved cardiac autonomic function, as indicated by an increased HRV, may contribute to the survival benefit from spironolactone treatment seen in previous studies in HD patients (2, 3). However, an increase in PVCs was observed and could indicate a possibly harmful effect with an increased risk of arrhythmias. These findings mandate cautious dosing of spironolactone or LTECG monitoring in subgroups at especially high risk of arrhythmia or sudden cardiac death.

## Supplementary Material

Effects of spironolactone on extrasystoles and heart rate variability in haemodialysis patients: a randomised crossover trial

Effects of spironolactone on extrasystoles and heart rate variability in haemodialysis patients: a randomised crossover trial
