# Supplementary material for: Effects of spironolactone on extrasystoles and heart rate variability in haemodialysis patients: a randomised crossover trial
Source: Ups J Med Sci. 2021 Jan 25;126:10.48101/ujms.v126.5660. doi: 10.48101/ujms.v126.5660 (PMC7886278; doi:10.48101/ujms.v126.5660)
Supplement: Effects of spironolactone on extrasystoles and heart rate variability in haemodialysis patients: a randomised crossover trial [file UJMS-126-5660-s001.pdf]

Supplementary material for: Eklund, M., Hellberg, O., Furuland, H., Cao, Y., & Nilsson, E. (2021). Effects of spironolactone on extrasystoles and heart rate variability in haemodialysis patients: a randomised crossover trial. Upsala Journal of Medical Sciences, 126. https://doi.org/10.48101/ujms.v126.5660

Supplemental table A. Concomitant medications.

Pharmacological agent	Number	%	Pharmacological agent	Number	%
Antidiabetics	*6*	*38*	*Drugs for CKD-MBD*	*16*	*100*
Insulin and analogues	5	31	CBPB	9	56
Sulfonylureas	1	6.2	Cinacalcet	2	12
			Lanthanum carbonate	2	12
Antithrombotic agents	*8*	*50*	Paracalcitol	1	6.2
Antiplatelet drugs	6	38	Sevelamer	10	62
Vitamin K antagonists	2	12	Vitamin D analogues	11	69
			Other	1	6.2
Cardiovascular drugs	*12*	*75*			
Beta blockers	8	50	*Potassium regulating drugs*	*3*	*19*
K^+ channel blockers	0	0	Polystyrene sulfonate	2	12
ACEi or ARB	8	50	Potassium chloride	1	6.2
CCB, vascular selective	3	19			
Organic nitrates	3	19	*Lipid modifying agents*	*8*	*50*
Other antihypertensive	1	6.2	Statins	8	50
			Bile acid sequestrants	0	0
Diuretics	*7*	*44*	Other	0	0
Loop diuretics	7	44			
Thiazides	0	0			

Concomitant medications in the 16 persons included in the long-term electrocardiogram analysis. Abbreviations: ACEi, Angiotensin converting enzyme inhibitor; ARB, Angiotensin receptor blocker; CBPB, Calcium based phosphate binder; CCB, Calcium channel blocker; CKD-MBD, Chronic kidney disease-mineral and bone disorder; K^+, Potassium.
