# Supplementary material for: Effects of spironolactone on extrasystoles and heart rate variability in haemodialysis patients: a randomised crossover trial
Source: Ups J Med Sci. 2021 Jan 25;126:10.48101/ujms.v126.5660. doi: 10.48101/ujms.v126.5660 (PMC7886278; doi:10.48101/ujms.v126.5660)
Supplement: Effects of spironolactone on extrasystoles and heart rate variability in haemodialysis patients: a randomised crossover trial [file UJMS-126-5660-s002.pdf]

Supplementary material for: Eklund, M., Hellberg, O., Furuland, H., Cao, Y., & Nilsson, E. (2021). Effects of spironolactone on extrasystoles and heart rate variability in haemodialysis patients: a randomised crossover trial. Upsala Journal of Medical Sciences, 126. https://doi.org/10.48101/ujms.v126.5660

Supplemental table B. Effect of spironolactone on atrial premature contractions, electrolytes and blood pressure.

Variable	Estimate	95% CI	*p*-value
Potassium, mmol/L	-0.046	-0.57 – 0.47	0.85
Sodium, mmol/L	-0.0093	-1.8 – 1.7	0.99
Magnesium[1]	0.96	0.9 – 1	0.18
Bicarbonate, mmol/L	-2	-3.5 – -0.52	0.013
APC[1]	2.5	0.58 – 11	0.2
Heart rate, min^{-1}	-2.1	-5.7 – 1.5	0.24
SBP, mmHg	13	-1.8 – 28	0.079
DBP, mmHg	5.6	-1.1 – 12	0.092

Estimates of the treatment effect of spironolactone, corresponding to absolute change or ratio between treatment and observation, for the respective parameters. The estimates, confidence intervals and *p*-values were calculated using a generalised linear mixed model with random effects, using treatment, intervention order and baseline values as covariates and treating patient effects as random. Magnesium and APC values were log-transformed, and estimates and confidence intervals were then antilogged to get a ratio between treatment and observation. The other variables were not transformed, and the estimates and Cls displayed represent absolute differences between treatment and observation. Abbreviations: APC, Atrial premature contraction; CI, Confidence interval; DBP, Diastolic blood pressure; SBP, Systolic blood pressure.

[1]Log-transformed before statistical analysis, antilogged ratios between treatment and observation are presented.
